# IL-33 Enhanced the Proliferation and Constitutive Production of IL-13 and IL-5 by Fibrocytes

**DOI:** 10.1155/2014/738625

**Published:** 2014-04-13

**Authors:** Hisako Hayashi, Akiko Kawakita, Shintaro Okazaki, Hiroki Murai, Motoko Yasutomi, Yusei Ohshima

**Affiliations:** Department of Pediatrics, Faculty of Medical Sciences, University of Fukui, 23-3 Shimoaizuki, Matsuoka, Yoshida-gun, Fukui 910-1193, Japan

## Abstract

Interleukin-33 appears to play important roles in the induction of allergic airway inflammation. However, whether IL-33 is involved in airway remodeling remains unclear. Because fibrocytes contribute to tissue remodeling in the setting of chronic inflammation, we examined the effects of IL-33 on fibrocyte functions. Fibrocytes were generated *in vitro* from peripheral blood mononuclear cells by culturing in the presence of platelet derived growth factors and the cells were stimulated with IL-33. IL-33 enhanced cell proliferation, *α*-SMA expression, and pro-MMP-9 activity by the fibrocytes without increasing endogenous transforming growth factor-*β*1 production. Fibrocytes constitutively expressed IL-13 and IL-5, and their production was augmented by stimulation with IL-33. Dexamethasone inhibited the functions of fibrocytes, but IL-33 made fibrocytes slightly refractory to the inhibitory effect of dexamethasone in terms of IL-13 production. Montelukast suppressed IL-13 production by nonstimulated fibrocytes but not those stimulated by IL-33. These findings suggest that IL-33 is involved in the airway remodeling process through its modulation of fibrocyte function independent of antigen stimulation. IL-33 might partially reduce the therapeutic effects of glucocorticoid and cysteinyl leukotriene receptor antagonist on fibrocyte-mediated Th2 responses.

## 1. Introduction

Fibrocytes are unique bone marrow-derived mesenchymal progenitor cells that are characterized by expression of hematopoietic cell markers (CD34, CD45, and leukocyte specific protein-1) and stromal cell markers (collagen I and proly-4-hydroxylase) [1–3]. Fibrocytes are presumably derived from a subpopulation of CD14^+^ peripheral blood monocytes [4]. Fibrocytes are involved in chronic inflammation, tissue repair, and fibrosis and might be a significant source of lung fibroblasts and myofibroblasts in response to lung injury and in remodeling of the airway wall [1, 5–8]. They can differentiate into *α*-smooth muscle actin (*α*-SMA)^+^ myofibroblasts and contribute to extracellular matrix generation leading to subepithelial fibrosis, airway basement membrane thickness [7, 9]. Recent data obtained from clinical settings suggest that the number of circulating fibrocytes might be a biomarker for disease progression in chronic lung diseases including asthma [10, 11].

Epithelial damage is one of the pathophysiological features observed in asthma. It is presumed that damage to the bronchial epithelium results in release of cytokines and growth factors that affect innate and adaptive immunity to allergen [12]. Because fibrocytes preferentially infiltrate the subepithelial zone in asthma, factors released from the bronchial epithelium may promote the airway recruitment and proliferation of fibrocytes and might modulate fibrocyte function [7, 9, 13].

IL-33 is a member of the IL-1 cytokine family. It induces adoptive Th2 immunity and signals through a complex that includes membrane-bound ST2 protein (ST2L) and IL-1 receptor accessory protein [14–16]. The IL-33/ST2L signaling pathway has been implicated in allergen-induced airway inflammation and hyperresponsiveness. IL-33 protein was elevated in bronchoalveolar lavage fluids of moderate asthmatics compared to mild and normal subjects. Moreover, increased airway epithelial IL-33 protein expression was found in severe asthma [17]. Recently, IL-33 has been shown to induce the production of Th2 cytokines IL-5 and IL-13 from innate immune cells without antigen stimulation and thereby might contribute to allergic pulmonary inflammation [18–20].

Although IL-33 supposedly contributes to airway remodeling by initiating Th2-mediated airway inflammation and by enhancing proliferation and differentiation of vascular endothelial cells [14], it is uncertain whether IL-33 exacerbate airway remodeling independent of the adaptive immune response. Therefore, in this study we examined the effects of IL-33 on the proliferation and function of fibrocytes. We show that IL-33 might play an important role in airway remodeling through innate immune responses.

## 2. Materials and Methods

### 2.1. Cell Culture

Peripheral blood was obtained from healthy volunteers with their written informed consent. The study was approved by the ethics committees of the University of Fukui. Peripheral blood mononuclear cells (PBMCs) (1.5 × 10^7^) isolated by Ficoll-Paque Plus (GE Healthcare Biosciences, Piscataway, NJ) were suspended in two mL of RPMI1640 + 10% fetal bovine serum (FBS) + penicillin and streptomycin (Invitrogen Co. Carlsbad, CA) and cultured in fibronectin-coated, six-well plates at 37°C with 5% CO_2_ for 72 h. Nonadherent cells were washed out over time with periodical changes of medium. To maximize the fibrocyte yield and the purity of adherent cells, 10 ng/mL of platelet derived growth factor-BB (PeproTec Inc., Rocky Hill, NJ) was added on day 7, as described previously [21]. After 2 weeks of culture, fibrocytes were harvested and then washed twice. The purity of the* in vitro* generated fibrocytes was 95% or greater as determined by flow cytometry [21]. Cells (1.2 × 10^5^ cells, 0.6 × 10^5^ cells, and 0.3 × 10^5^cells) were cultured in FBS-free RPIM1640 in fibronectin-coated 12-, 24-, and 96-well plates, respectively. Medium was replaced the next day with fresh FBS-free RPIM1640. The cells were stimulated with IL-33 (PeproTec Inc.) in the presence or absence of dexamethasone (Sigma-Aldrich), montelukast (Merck & Co. Inc., Whitehouse Station, NJ), or anti-TGF-*β*1 antibody (R&D Systems Inc., Minneapolis, MN). After 72 h of stimulation, cell proliferation was assessed by a colorimetric cell viability assay or a BrdU incorporation assay using a Cell Counting Kit-8 (Dojindo, Kumamoto, Japan) or a Cell Proliferation Kit (GE Healthcare Life Sciences, Uppsala, Sweden), respectively. Assays were done in triplicate wells. Relative viable cell numbers were determined by dividing the absorbance at 450 nm of stimulated fibrocytes by that of nontreated control fibrocytes.

### 2.2. RNA Isolation and RT-PCR

After a 24 h culture with stimulation, total RNA was isolated using an RNeasy Mini Kit (QIAGEN K.K., Tokyo, Japan). First-strand cDNA was synthesized from 0.5 *μ*g of total RNA using the Superscript II First Strand System (Invitrogen Co.). Polymerase chain reaction (PCR) amplification of the ST2L gene and the soluble splicing variant ST2L (sST2) gene was carried out using specific primers. The primer sequences for ST2L were as follows: CTGTCTGGCCCTGAATTTGC (sense) and AGCAGAGTGGCCTCAATCCA (antisense). The primer sequences for sST2L were as follows: CTGTCTGGCCCTGAATTTGS (sense) and TGGAACCACACTCCATTCTGC (antisense). PCR amplification of the housekeeping gene *β*-actin was performed for each sample for normalization between samples.

### 2.3. Flow Cytometric Analysis

Cells were pretreated with BD Cytofix/Cytoperm (BD Biosciences, San Diego, CA) and then incubated with anti-*α*-SMA monoclonal antibody (mAb) (Sigma-Aldrich) or isotype control IgG, along with human IgG in BD Perm/Wash (BD Biosciences). After washing, FITC-conjugated F(ab*'*)_2_ of goat anti-rabbit IgG (Rockland, Gilbertsville, PA) was added. Specific mean fluorescence intensities (ΔMFI) were determined by subtracting the isotype-matched control antibody fluorescence.

### 2.4. Intracytoplasmic Cytokine Staining

In order to enhance intracellular cytokine staining signals by blocking protein transport processes, cells were cultured in the presence of monensin (GolgiStop, BD Biosciences) for 3 h. The cells were fixed and permeabilized with −20°C 100% ethanol and then blocked for one hour with phosphate-buffered saline—0.05% Tween 20, containing normal human IgG. The cells were stained with PE-conjugated anti-IL-13 antibody (BD Biosciences) or isotype control IgG. The images were obtained using Olympus IX70 inverted microscopes (Olympus Co., Tokyo, Japan).

### 2.5. Gelatin Zymography

Culture supernatants were resolved by 7.5% SDS-PAGE in the presence of one mg/mL gelatin. After electrophoresis, gels were washed for 30 min in 2.5% Triton-X-100 at room temperature to remove SDS. Gels were then washed for 30 min and incubated overnight at 37°C in reaction buffer (50 mM Tris-HCl, 0.2 M NaCl, 5 mM CaCl_2_, 0.02% Brij35, and pH 7.6). After staining with Coomassie Brilliant Blue R-250, gelatin-degrading activities of matrix metalloproteinases (MMPs) were identified as clear zones of lysis against a blue background. Gelatin zymography standard was obtained from Millipore. Photographs of the zymograms were scanned using a LAS-3000 mini (Fuji Film, Tokyo, Japan), and the intensity of the digitalized bands was analyzed by Multi Gauge Version 3.0 (Fuji Film).

### 2.6. Cytokine Measurements

IL-5 and IL-13 concentrations were determined by two-site sandwich ELISAs, using the protocols recommended by the antibody suppliers. Antibody pairs and standard recombinant cytokines for ELISA were purchased from Thermo Fischer Scientific Inc. (Rockford, IL). TGF-*β*1 levels were determined with ELISA kits (Invitrogen Co.).

### 2.7. Statistical Analysis

Comparisons of two groups used unpaired Student's *t*-tests, unless an *F*-test showed that the variances were significantly different. When variances were significantly different, Welch's test or paired Student's *t*-test was used. A *P* value less than 0.05 denoted a statistically significant difference.

## 3. Results

### 3.1. Fibrocytes Generated from PBMCs Expressed ST2L and sST2

As previously reported [21], the fibrocytes generated from PBMCs expressed *α*-SMA, calponin, collagen type I, and trace amounts of CD34, consistent with the typical phenotype of* in vitro* cultured fibrocytes, but did not express other cell lineage markers (i.e., dendritic cells; CD1 and CD83 and monocytes; CD14 and endothelial cells; von Willebrand factor and T cells; CD3 and B cells; CD19) (data not shown) [1].

**Figure 1:**
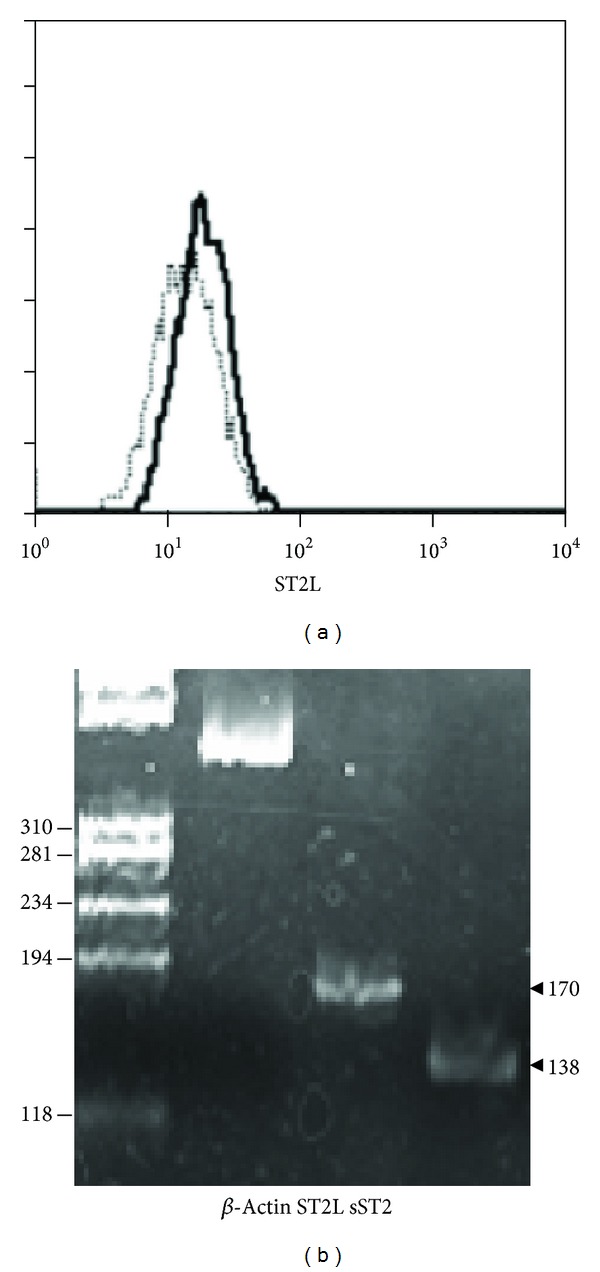
ST2L and sST2 mRNA and protein expression by fibrocytes generated* in vitro* from PBMC. (a) Cell surface expression of ST2L protein on fibrocytes was detected by flow cytometry. Cells were stained with anti-ST2/IL-1R4 mAb (thick line) or isotype control IgG (thin line). (b) ST2L and sST2 mRNA expression was determined by RT- PCR.

It has been shown that circulating peripheral blood fibrocytes expressed ST2L [22]. Consistent with that finding, the fibrocytes generated from PBMC* in vitro *also expressed ST2L in mRNA and protein levels (Figure 1). The fibrocytes also expressed mRNA for sST2, another product of ST2 gene as a result of alternative splicing, which is presumed to acts as a decoy receptor to prevent IL-33 binding to and signaling through sT2L [14].

### 3.2. IL-33 Enhanced Fibrocyte Proliferation

**Figure 2:**
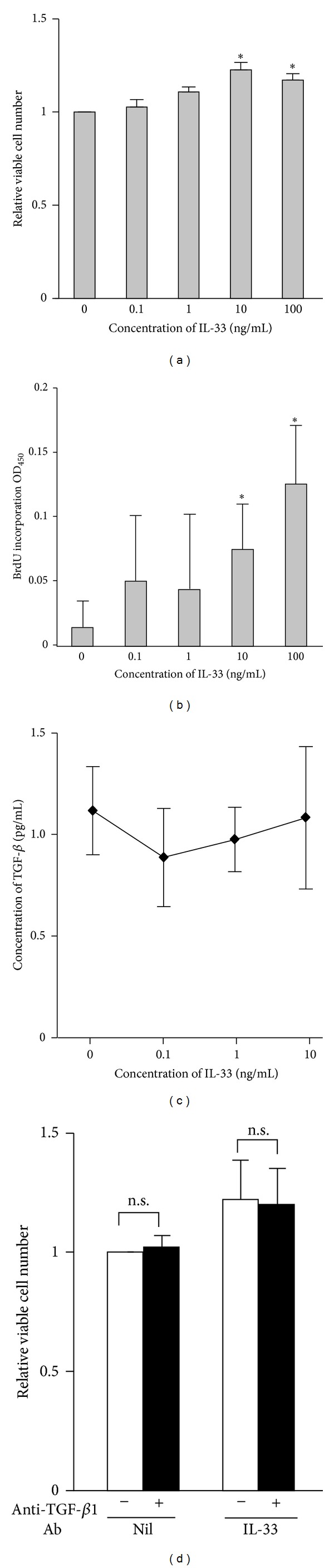
Effects of IL-33 on fibrocyte proliferation and endogenous TGF-*β*1 production. Fibrocytes were cultured in the presence of various concentrations of IL-33. After 72 h of culture, relative viable cell number and DNA synthesis were determined by a colorimetric cell viability assay (a) and a BrdU incorporation assay (b), respectively. The concentration of TGF-*β*1 in the supernatants was measured by ELISA (c). In (d), the relative proliferation of fibrocytes stimulated with or without 10 ng/mL IL-33 in the presence (closed bars) or absence (open bars) of 20 *μ*g/mL anti-TGF-*β*1 antibody was analyzed by a colorimetric assay. The data are expressed as means ± SEM (*n* = 4). *P < 0.05, n.s.: not significant.

Using a colorimetric assay, we observed that the relative number of viable fibrocytes generated* in vitro* was significantly increased in an IL-33 concentration-dependent manner, compared with nonstimulated control fibrocytes (Figure 2(a)). A BrdU incorporation assay confirmed that* in vitro* generated fibrocytes proliferated little, if any. However, IL-33 enhanced their proliferation at concentrations ≥10 ng/mL (Figure 2(b)). Since TGF-*β*1 is profibrogenic growth factor and well known to induce fibrocyte differentiation [4], we asked whether the mitogenic effect of IL-33 depended on endogenously produced TGF-*β*1. IL-33 did not increase TGF-*β*1 production and neutralizing TGF-*β*1 antibody did not suppress fibrocyte proliferation with or without IL-33 stimulation, indicating that the proliferation of* in vitro* generated fibrocytes was not attributable to endogenous TGF-*β*1 (Figures 2(c) and 2(d)).

### 3.3. IL-33 Enhanced *α*-SMA Expression and Pro-MMM-9 Activity

**Figure 3:**
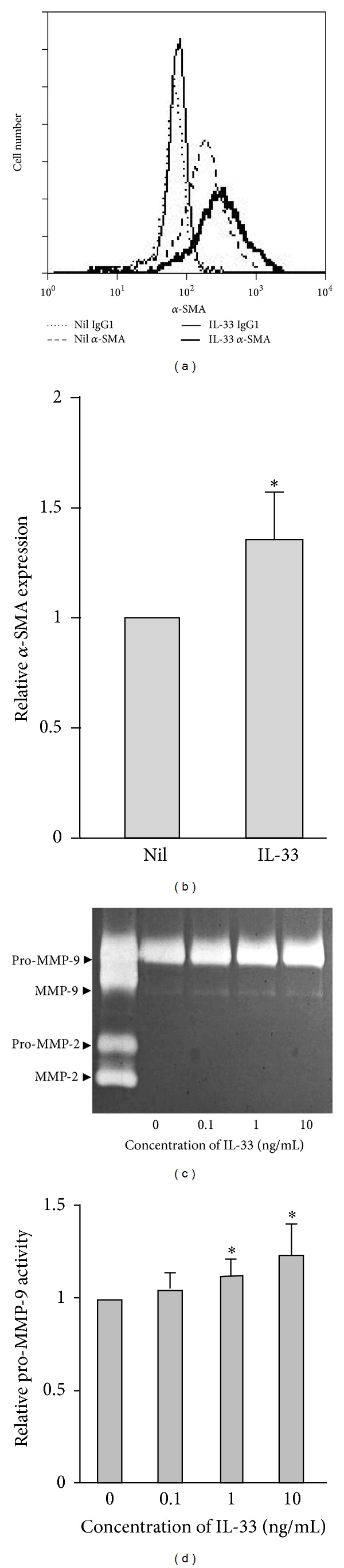
IL-33 enhanced *α*-smooth muscle actin expression and pro-MMP-9 activity. Fibrocytes were cultured with (solid lines) or without (broken lines) 10 ng/mL IL-33 for 72 h. Cells were stained with anti-*α*-SMA mAb (thick line) or isotype control IgG (thin line) (a). Relative expression of *α*-SMA with and without IL-33 was determined by flow cytometry (b). The gelatinase activities of MMP-2 and MMP-9 were analyzed by gelatin zymography (c) and its relative expression was determined by densitometry (d). Results are means ± SEM (*n* = 4). *P < 0.05.

Fibrocytes are presumed to migrate into injured tissue by using MMPs and then transdifferentiate into myofibroblasts with the enhanced expression of *α*-SMA [4, 23]. IL-33 increased the constitutive expression of *α*-SMA protein, indicating that it induced transdifferentiation of fibrocytes (Figures 3(a) and 3(b)). Monocyte-derived fibrocytes are known to express various MMPs, including MMP-2 and MMP-9 [23, 24]. To evaluate the activity of MMP-2 and MMP-9 produced by IL-33-stimulated fibrocytes, conditioned media were analyzed by gelatin zymography. As shown in Figures 3(c) and 3(d), gelatinolytic bands corresponding to MMP-9 and pro-MMP-9 were revealed, whereas those of MMP-2 and pro-MMP-2 were below detection. The fibrocytes stimulated by IL-33 expressed increased gelatinolytic activity of pro-MMP-9 depending on the concentration of IL-33.

### 3.4. IL-33 Enhanced Constitutive IL-13 and IL-5 Production from Fibrocytes

**Figure 4:**
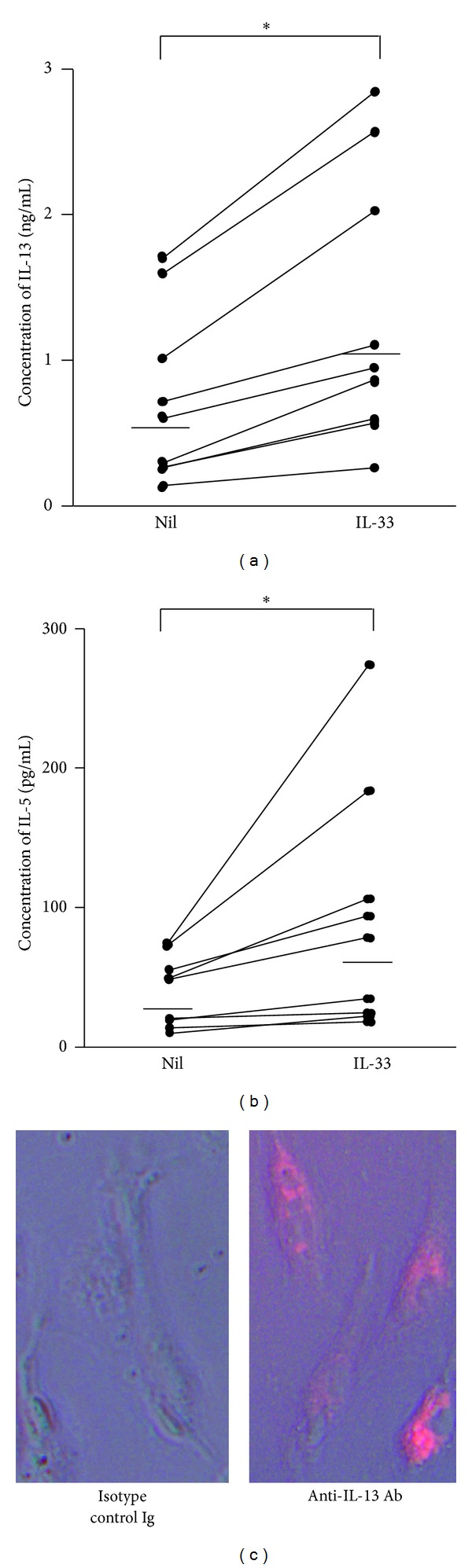
IL-33 increased IL-13 and IL-5 production by fibrocytes. Fibrocytes were stimulated with 10 ng/mL of IL-33 for 72 h. IL-13 (a) and IL-5 (b) levels in the supernatants were measured by ELISA. Horizontal thick bars represent the median of each group. Fibrocytes cultured in the presence of GolgiStop during the last three h of culture were stained with anti-IL-13 antibody (right panel) or isotype control IgG (left panel) (c). Intracytoplasmic IL-13 was detected by immunofluorescent microscopy. *P < 0.05.

We next examined whether fibrocytes were able to produce these type 2 cytokines without antigen stimulation. It is of note that fibrocytes constitutively produced IL-13 and IL-5, and 10 ng/mL IL-33 enhanced productions of these cytokines (Figures 4(a) and 4(b)). To further corroborate the constitutive production of IL-13 from fibrocytes by themselves without either antigen or IL-33 stimulation, cells were cultured in the presence of monensin, an intracellular protein transport inhibitor, after which intracellular accumulation of IL-13 was assessed by immunofluorescence staining. As illustrated in Figure 4(c), immunofluorescence microscopy revealed the accumulation of constitutively produced IL-13 in spindle-shaped cells corresponding to fibrocytes.

### 3.5. Dexamethasone Inhibited Proliferation, Cytokine Production, and Pro-MMP9 Activity

**Figure 5:**
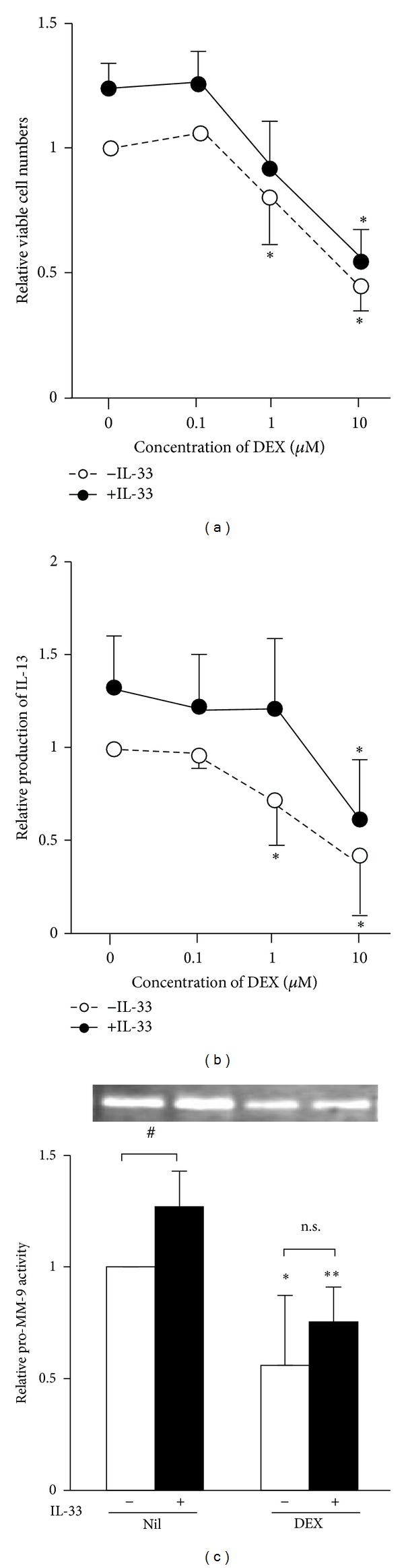
Effects of dexamethasone on cell proliferation, expression of IL-13, and pro-MMP-9 activity. Fibrocytes were cultured with (closed circles and bars) or without (open circles and bars) 10 ng/mL IL-33 for 72 h in the presence of various concentrations (a) and (b) or 10 *μ*M (c) dexamethasone. Relative proliferation (a), IL-13 production (b), and pro-MMP-9 activity (c) were analyzed by colorimetric cell viability assays, ELISA, and gelatin zymography, respectively. Results are means ± SEM (*n* = 6). *P < 0.05, **P < 0.01 (compared with 0 *μ*M dexamethasone), ^#^ *P* < 0.05, n.s.: not significant.

The possible therapeutic effects of glucocorticoids on airway remodeling remain controversial. Dexamethasone (DEX) inhibited the proliferation of both nonstimulated and IL-33-stimulated fibrocytes in a dose dependent manner (Figure 5(a)).DEX did not affect the expression levels of ST2L and sST2 mRNA (data not shown), suggesting that DEX did not interfere with IL-33 signaling at the receptor level. IL-13 production by nonstimulated fibrocytes was suppressed by ≥1 *μ*M DEX, whereas that by IL-33-stimulated fibrocytes was suppressed by only 10 *μ*M DEX (Figure 5(b)). One *μ*M DEX suppressed IL-13 production by nonstimulated and IL-33-stimulated fibrocytes 32.1 ± 6.8 and 8.0 ± 7.2% (Mann-Whitney *U* test; *P* < 0.05), respectively. Thus IL-33 stimulation might make fibrocytes relatively steroid resistant. 10 *μ*M DEX suppressed pro-MMP-9 activity expressed by both nonstimulated and IL-33-stimulated fibrocytes (Figure 3(a)).

### 3.6. IL-33 Stimulation Made Fibrocytes Refractory to the Inhibitory Effects of Cysteinyl Leukotriene Receptor 1 Antagonists

**Figure 6:**
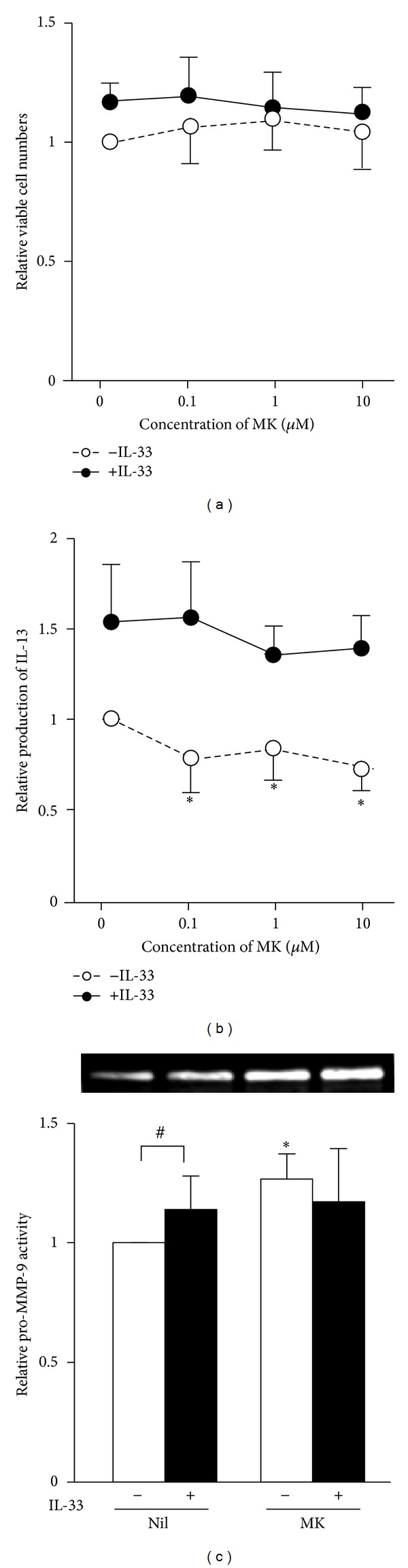
Effects of montelukast on cell proliferation, expression of IL-13, and pro-MMP-9 activity. Fibrocytes were cultured with (closed circles and bars) or without (open circles and bars) 10 ng/mL IL-33 for 72 h in the presence of various concentrations (a) and (b) or 10 *μ*M (c) montelukast (MK). Relative proliferation (a), IL-13 production (b), and pro-MMP-9 activity (c) were analyzed by colorimetric cell viability assays, ELISA, and gelatin zymography, respectively. Results are means ± SEM (*n* = 6). *P < 0.05 (compared with 0 *μ*M MK), ^#^ *P* < 0.05, n.s.: not significant.

Cysteinyl leukotrienes are key mediators of the airway remodeling process [25]. Both murine and human fibrocytes express both CysLT1 and CysLT2 [26]. CysLT1 antagonists inhibit exogenous LTD_4_-induced proliferation of murine fibrocytes. Since murine fibrocytes are capable of producing cysteinyl leukotrienes [27], we examined the effects of a CysLT1 antagonist, montelukast, on human fibrocyte function. Montelukast did not affect the expression levels of ST2L and sST2 mRNA (data not shown). As shown in Figure 6, montelukast did not inhibit proliferation but significantly suppressed constitutive IL-13 production and enhanced pro-MMP-9 activity expressed by human fibrocytes. The inhibitory effect of montelukast on IL-33-induced IL-13 production was not observed.

## 4. Discussion

In the present study, we have demonstrated that IL-33 induced the proliferation of fibrocytes generated* in vitro *and allowed these cells to differentiate into a myofibroblastic phenotype. More intriguingly, the fibrocytes were able to constitutively produce type 2 cytokines, and IL-33 augmented the production of IL-13, a profibrogenic cytokine even without antigen stimulation. These data collectively suggest that IL-33 plays an important role in fibrocyte-mediated tissue remodeling.

Recently, Bianchetti et al. reported that IL-33 does not induce proliferation of circulating fibrocytes isolated from nonasthmatics but does enhance the proliferation of fibrocytes from patients with allergen-exacerbated asthma [22]. The difference in the responsiveness to IL-33 in terms of cell proliferation may be explained by the maturation stage of fibrocytes. It has been reported that downregulation of CD34 and CD45RO expression occurs in parallel with an increased expression of *α*-SMA as fibrocytes differentiate and mature [7, 26]. Circulating fibrocytes robustly express CD34, whereas the fibrocytes generated* in vitro* express trace amounts of CD34 and large amounts of *α*-SMA [21, 22]. Airway smooth muscle cell-derived PDGF has been shown to promote fibrocyte migration to smooth muscle bundles* in vitro *[28]. In this context, the fibrocytes generated* in vitro* by culturing in the presence of PDGF are maturer than circulating fibrocytes and might correspond to tissue resident fibrocytes recruited in the airway smooth muscle compartment in asthma.

Human CD34^+^ cells isolated from peripheral blood and cord blood express ST2L and respond to IL-33* ex vivo* by secreting large quantities of Th2 cytokines [18]. Moreover, CD34^+^ cells producing IL-5 and IL-13 are detected in the sputum of individuals with allergic asthma. Around 25% of fibroblasts-like cells in BAL fluid from asthma patients with basement membrane thickness express fibrocytes markers CD34 and *α*-SMA [9]. Basement membrane thickness is correlated to the number of fibrocytes in tissue. Given that circulating CD34^+^ cells are comprised of heterogeneous cell populations, fibrocytes could be important sources of IL-13 and IL-5 in BAL fluid and airway tissues of asthmatics. Because IL-13 is an important cytokine involved in airway remodeling in asthma, IL-33 might augment airway remodeling through its enhancement of IL-13 production from fibrocytes without allergen stimulation [29, 30].

Bronchial epithelial cells of asthmatic patients express elevated levels of thymic stromal lymphopoietin (TSLP) as well as IL-33 [17, 30]. TSLP induces bronchial epithelial cell proliferation and increases injury repair through IL-13 [30]. TSLP expression of epithelial cells is enhanced by stimulation with IL-13 [31]. In this context, crosstalk between epithelial cells and fibrocytes via the IL-33/IL-13 axis might play an important role in airway remodeling in concert with TSLP.

Fibrocytes have been shown to produce and secrete MMP-2, MMP-7, MMP-8, and MMP-9 [23]. MMP-9 dissolves several extracellular matrix proteins and is implicated in the proteolysis of the basement membrane during the early invasion stage of angiogenesis as well as the migration of fibrocytes from the circulation to the tissues [23, 24]. Moreover, MMP-9 can activate latent TGF-*β*, a pleiotropic growth factor that directly induces a variety of responses associated with lung fibrosis and airway remodeling [32, 33]. Therefore, IL-33 might facilitate angiogenesis and fibrosis in airway remodeling by enhancing pro-MMP-9 activity from fibrocytes.

We have previously reported that glucocorticoid suppresses cytokine production and upregulation of *α*-SMA expression by activated fibrocytes [21]. Similarly, dexamethasone inhibited fibrocytes' proliferation and their expression of pro-MMP-9, which governs local accumulation of fibrocytes. In patients with chronic kidney diseases, it has been shown that the number of interstitial fibrocytes was significantly decreased by glucocorticoid therapy [34]. In this context, glucocorticoid may effectively prevent fibrocytes from accumulating at the sites of airway remodeling.

The inhibitory effect of montelukast on IL-13 production by nonstimulated fibrocytes was not due to cytotoxicity because IL-33-stimulated fibrocytes were refractory to the inhibitory effect of montelukast even at high concentrations. Endogenous cysteinyl leukotrienes released by fibrocytes might regulate constitutive IL-13 production in an autocrine fashion. Montelukast, but not DEX, was shown to reverse established features of airway remodeling, including subepithelial fibrosis in a chronic asthma mouse model [27]. Montelukast-mediated enhancement of pro-MMP-9 activity by fibrocytes might be involved in the reduction of subepithelial collagen deposition.

IL-33 and TNF-*α* expression in lung tissues from asthmatic subjects increases with the severity of asthma [35]. Dexamethasone fails to abolish TNF-*α*-induced IL-33 upregulation in airway smooth muscle cells, which are a major source of IL-33 in asthma. We found that montelukast failed to suppress IL-13 production by IL-33-stimulated fibrocytes and that higher concentrations of dexamethasone were required to suppress it compared with nonstimulated fibrocytes, suggesting that IL-33 might partially reduce the therapeutic effects of glucocorticoid and cysteinyl leukotriene receptor antagonist on fibrocyte-mediated Th2 responses.

## 5. Conclusions

In conclusion, the IL-33-fibrocytes type 2 cytokine axis may account for the airway remodeling associated with allergic inflammation regardless of allergen-specific immune responses. IL-33 might play a pivotal role in the pathophysiology of refractory asthma characterized by persistent symptoms despite use of multiple regular asthma therapies, including inhaled corticosteroids and CysLTR antagonist.
